# CMR in the diagnosis of acute pericarditis

**DOI:** 10.1186/1532-429X-13-S1-O35

**Published:** 2011-02-02

**Authors:** Nicholas J Brett, Damian Roper, Richard E Slaughter, Christian Hamilton-Craig

**Affiliations:** 1 The Prince Charles Hospital, Chermside, Australia

## Background

Acute pericarditis is the most common condition affecting the pericardium. Diagnosis has historically been based upon a combination of clinical history, examination, ECG findings, inflammatory markers and echocardiographic findings of pericardial thickening and effusion. Literature on the role of CMR and the appearances of acute pericarditis is limited.

## Method

All patients with a clinical diagnosis of acute pericarditis and who had a cardiovascular magnetic resonance (CMR) examination from January 2006 to June 2010 were retrospectively evaluated from a high-volume center (performing 6,230 CMR studies during this period) Patients with confirmed myocarditis or myocardial infarction, or previous cardiac surgery were excluded. Age and sex matched controls were obtained from the department’s database.

Images were blindly evaluated by an expert radiologist with SCMR level 3 experience. Pericardial thickness was measured on T2 black blood DIR images to minimize artifact from chemical shift. Pericardial enhancement on late gadolinium enhancement (LGE) images was graded from 0-3 (0=absent, 3=intense enhancement). Pericardial effusions were graded as physiological (trivial/small) vs pathological (medium or large). The presence of pleural effusions was also recorded.

## Results

CMR images from 21 patients with clinically confirmed acute pericarditis were reviewed. Pericardial thickness was significantly increased in patients with pericarditis compared with controls 2.35 +/- 0.54mm vs 1.77 +/- 0.34mm (p<0.05). Pericardial LGE was demonstrated in 19 (86%). Of these, 12 (63%) had intense, 5 (26%) moderate, and 1 (5)% mild LGE. In comparison no controls had significant LGE. There was a strong correlation between the presence of LGE and the clinical diagnosis of acute pericarditis (p<0.01). No patient had myocardial LGE.

**Figure 1:**
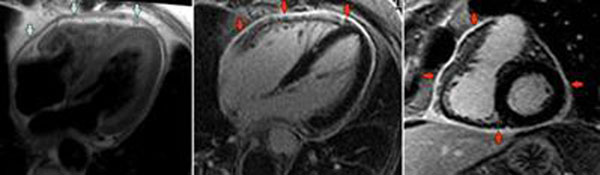
4 chamber DIR image demonstrating pericardial thickening (left) and 4 chamber (centre) and short axis LGE images (right) demonstrating intense pericardial enhancement

Pericardial effusion was present in 13 (62%) of the patients with acute pericarditis. Of these, 3 (23%) had a moderate size pericardial effusion and 10 (77%) had small/trivial effusions. No control had pathological pericardial effusion. Pleural effusions were significantly more common in patients with pericarditis (43% vs 0%, p<0.001). Figure 1.

## Discussion

Presence of pericardial LGE, particularly moderate or intense enhancement, is strongly suggestive of acute pericarditis. Pericardial effusions were generally small, and pericardial thickening was mildly but significantly increased compared to controls. Pleural effusions were present in 46% of patients with pericarditis, suggesting a generalized polyserositis. CMR is useful in the diagnosis of acute pericarditis, enabling confirmation of the diagnosis and adding value over-and-above standard-of-care investigations.

